# Numerical Simulation of Arc Welding in Large Flange Shafts Based on a Novel Combined Heat Source Model

**DOI:** 10.3390/ma18173932

**Published:** 2025-08-22

**Authors:** Zhiqiang Xu, Chaolong Yang, Wenzheng Liu, Ketong Liu, Feiting Shi, Zhifei Tan, Peng Cao, Di Wang

**Affiliations:** 1 School of Chemical and Machinery, Liaodong University, Dandong 118001, China; xuzhiqiang@liaodongu.edu.cn (Z.X.);; 2 College of Architecture and Civil Engineering, Beijing University of Technology, Beijing 100124, China; 3 College of Architecture and Civil Engineering, Xi’an University of Science and Technology, Xi’an 710054, China; 4 School of Civil Engineering, Yancheng Institute of Technology, Yancheng 224051, China; shifeiting@ycit.cn; 5 Department of Civil and Environmental Engineering, The Hong Kong Polytechnic University, Hung Hom, Hong Kong, China

**Keywords:** Fe-C-Mn-Cr low-alloy medium carbon steel, large flange shaft, combined heat source model, temperature field, deformation field, residual stress distribution

## Abstract

Welding, as a critical process for achieving permanent material joining through localized heating or pressure, is extensively applied in mechanical manufacturing and transportation industries, significantly enhancing the assembly efficiency of complex structures. However, the associated localized high temperatures and rapid cooling often induce uneven thermal expansion and contraction, leading to complex stress evolution and residual stress distributions that compromise dimensional accuracy and structural integrity. In this study, we propose a combined heat source model based on the geometric characteristics of the weld pool to simulate the arc welding process of large flange shafts made of Fe-C-Mn-Cr low-alloy medium carbon steel. Simulations were performed under different welding durations and shaft diameters, and the model was validated through experimental welding tests. The results demonstrate that the proposed model accurately predicts weld pool geometry (depth error of only 2.2%) and temperature field evolution. Meanwhile, experimental and simulated deformations are presented with 95% confidence intervals (95% CI), showing good agreement. Residual stresses were primarily concentrated in the weld and heat-affected zones, exhibiting a typical “increase–steady peak–decrease” distribution along the welding direction. A welding duration of 90 s effectively reduced residual stress differentials perpendicular to the welding direction by 19%, making it more suitable for medium carbon steel components of this scale. The close agreement between simulation and experimental data verifies the model’s reliability and indicates its potential applicability to the welding simulation of other large-scale critical components, thereby providing theoretical support for process optimization.

## 1. Introduction

Welding is a highly efficient and reliable process for permanent material joining, and its applications have permeated various fields, ranging from traditional mechanical manufacturing and construction engineering to emerging industries, such as aerospace and renewable energy, where it plays an irreplaceable role [1,2]. It has significant advantages over traditional mechanical connections and cutting processes [3]. However, the welding process involves complex metallurgical reactions and thermal cycles, which easily lead to problems such as residual stress, deformation, and uneven microstructure, thereby seriously affecting the service performance of welded joints [4]. Therefore, it is necessary to conduct in-depth research on the characteristics of welding processes so as to provide a basis for controlling the welding quality of structures and improving the reliability and service life of products.

Residual stresses exert a significant impact on the engineering performance of materials and structural components, particularly in terms of fatigue life, deformation, dimensional stability, corrosion resistance, and brittle fracture [5], as well as having a significant impact on the strength and other mechanical properties of materials [6]. For example, when a component with residual stresses is subjected to external loads, the total stress state always includes both the applied stress and the residual stress [7]. Residual stresses significantly affect the static and fatigue strength of components and play a crucial role in the initiation and propagation of cracks near welds [8,9,10]. Moreover, residual stresses can cause workpiece deformation as they attempt to rebalance after processing, thereby affecting dimensional accuracy and stability [11,12]. In addition, in the assessment of residual stresses in welded structures, the thermal history during the welding process cannot be ignored. The thermal history not only regulates the residual stress field through temperature gradients and cooling rates but also significantly affects the microstructural transformation of the joint. For instance, Alipooramirabad et al. [13] studied the relationship between the residual stress, microstructure, and mechanical properties in welded joints of high-strength low-alloy steel. They observed that residual stress exceeding the yield strength was generated on the upper surface of the welded joint, and both residual stress and hardness showed a decreasing trend with the increase in heat input. Rae et al. [14] analyzed the correlation between residual stress and microstructure evolution in electron beam welded joints of Ti-6Al-4V alloy, and their research found that the morphology of the α phase in the weld zone is closely related to the changes in residual stress. Therefore, it is necessary to comprehensively consider macroscopic factors and microstructural aspects to accurately evaluate the residual stress distribution in welded structures so as to fully reflect the quality of welded joints. This is of great significance for guiding the welding processes of large-scale industrial installations and ensuring the structural integrity of equipment.

Despite these efforts, accurately predicting welding-induced residual stress remains challenging due to the inherent complexity of the process [15]. Experimental testing and numerical simulation are the two common methods for studying welding residual stresses [16,17,18,19]. In experimental testing, the measurement of residual stress can be roughly divided into destructive methods and non-destructive methods. Destructive methods measure stress through strain relief, but they are destructive in nature and their results are susceptible to interference [20,21,22,23]. Non-destructive methods (such as XRD, neutron diffraction, and FIB-DIC) can achieve non-destructive testing [24]. Furthermore, recent studies have explored the use of active thermographic methods (such as step-heating laser thermography) for the non-contact detection of microstructural differences in welds, including changes in morphology and material response caused by different process parameters. Dell’Avvocato et al. [25] proposed a procedure for dissimilar aluminum joints obtained by P-FSSW, demonstrating the ability of thermographic parameters to distinguish weld morphologies and indirectly infer mechanical behaviors. By quickly locating the stress concentration zones in welded joints and conducting quantitative measurements in these zones using XRD or the blind hole method, both efficiency and accuracy are achieved. However, considering the limitations of the experimental measurement of residual stress, such as destructiveness, high costs, and difficulty in achieving accurate characterization of full-field/internal stress, the finite element method has become an effective tool for reproducing the welding process and predicting welding residual stress [26,27,28]. Murugan [29] employed a double-ellipsoid heat source model to simulate the 3D transient thermos-mechanical forces in T-joint welds and predict the longitudinal residual stress field. Meanwhile, the contour method was used to verify the residual stress obtained from the finite element simulation, and the experimental results were in good agreement with the finite element simulation results. Gadallah [30] calculated the residual stress of welds using the thermo-elastoplastic finite element method, taking into account the changes in volume and mechanical properties with temperature. The residual stresses, measured by the contour method and X-ray diffraction, were consistent with those calculated by the finite element method. Shi [31] combined optical technology with the contour method to measure the multi-directional residual stress in welded samples. Additionally, a Gaussian heat source model was used to perform a finite element nonlinear transient thermal analysis of the welding process, and the predicted residual stress obtained from the finite element simulation was consistent with that from the contour method. The above studies have verified the reliability of the finite element method.

In the simulation of welding processes using the finite element method, the heat source models are employed to describe the heat input. These heat source models serve as the foundation for obtaining temperature field distributions, and the accuracy of welding finite element simulations is largely influenced by the adopted heat source model [32]. Traditional heat source models include Gaussian distributed heat sources, double-ellipsoid heat sources, and uniform-volume heat sources, which are widely applied. However, when existing single-heat-source models are used for molten pool simulation, their results are often limited to a few specific molten pool shapes such as semi-ellipsoidal and conical, thereby reducing the simulation accuracy. The combination of multiple heat source models can not only accurately describe molten pools with complex shapes but also be applicable to numerical simulations of different welding methods. Currently, combined heat source models are mainly constructed based on two scenarios: molten pool shape and the heat distribution of the heat source [33]. Wu et al. [34] developed a combined heat source model tailored to the characteristics of plasma arc welding. This model integrates the features of a double ellipsoid and a quadratic cone, with its parameters dynamically adjusted according to changes in the keyhole depth during the welding process. The simulation results were found to be in good agreement with the experimental results. Through extensive finite element simulations of the welding process, the post-weld residual stress distribution can be analyzed quickly and comprehensively [35]. Meanwhile, combining a small number of welding tests to verify the finite element simulation results enables a more economical and effective study of welding phenomena [36]. This provides reliable support for optimizing welding process parameters, avoiding problems arising during welding and ensuring the stability of the welding process.

In light of previous studies, it is evident that welding—especially in heavy-load applications—faces considerable challenges, including deformation, residual stress, and weld pool morphology. These factors critically affect subsequent assembly, operational reliability, and fatigue life. This study is focused on the arc welding of large-diameter flanged shafts (40 mm and 60 mm) using Fe-C-Mn-Cr low-alloy medium carbon steel as the base material. A novel combined heat source model is developed, based on extracted weld pool geometry, to simulate and analyze the welding process. The validated model is then used to assess the effects of various welding parameters. The remainder of this paper is organized as follows. First, welding experiments on large flanged shafts are conducted under different process conditions, with real-time temperature and deformation data collected from key locations. Next, thermo-elastoplastic FEM simulations are performed using the proposed combined heat source model to replicate the welding process under realistic conditions. Finally, simulation and experimental results are compared to verify the accuracy of the heat source model, while discussing the thermal field, deformation field, and residual stress field under different process parameters.

## 2. Materials and Methods

### 2.1. Materials and Experimental Procedure

Fe-C-Mn-Cr low-alloy carbon steel, specifically 45Mn steel, is a widely used alloy known for its high strength, good plasticity, toughness, and excellent machinability. It also exhibits superior impact toughness and fatigue resistance [37,38]. Compared to high-alloy Cr-Ni steels, 45Mn steel is more cost-effective and easier to process, making it suitable for manufacturing load-bearing components such as shafts, crankshafts, connecting rods, bolts, splines, gears, and mold bases. The experimental specimens in this study were fabricated from Fe-C-Mn-Cr low-alloy medium carbon steel, with the base material supplied by Angang Steel Company Limited, Anshan, China. E5015 electrodes, manufactured by Tianjin Jin Qiao Welding Material Group Co., Ltd., Tianjin, China, were used for the welding process. The chemical compositions of both the base and filler materials are listed in Table 1. It can be seen that there are obvious differences in chemical composition between the base material and the filler material, with significant disparities in their thermal properties and mechanical properties. The base material conforms to the Chinese standard GB/T 711-2017 [39], while the electrodes meet the American Welding Society standard AWS A5.1/A5.1M-2012 [40].

**Figure 1:**
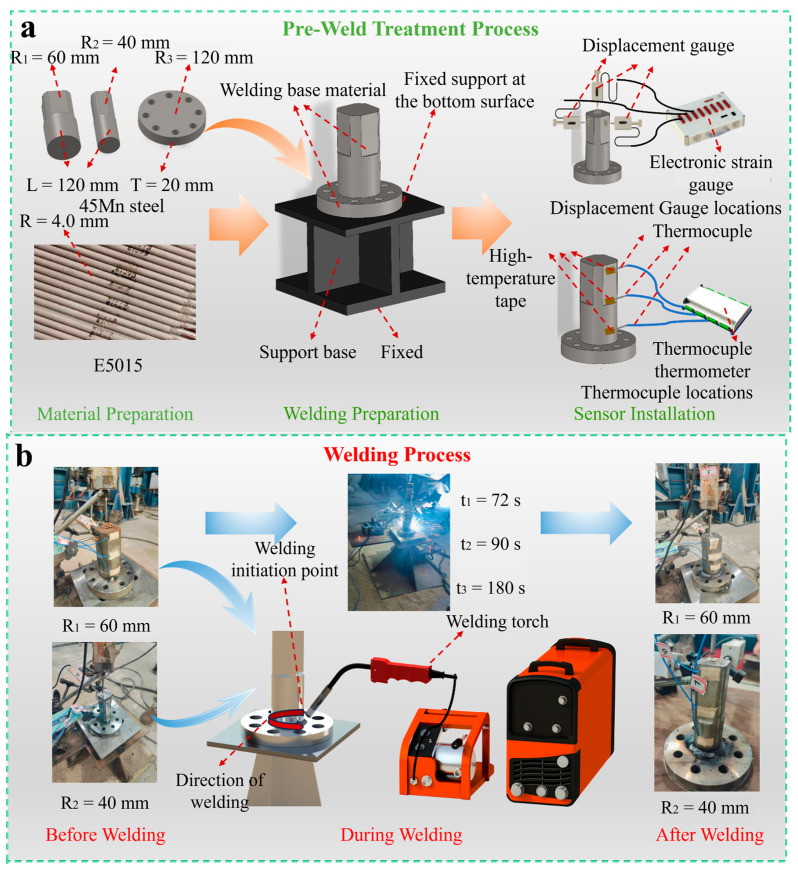

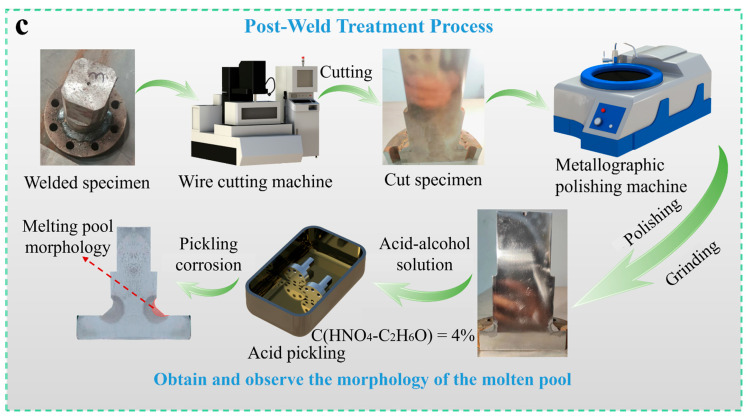
Material preparation and experimental process: (**a**) pre-welding treatment; (**b**) welding process; (**c**) post-welding treatment.

Figure 1 provides an overview of the experimental workflow, including pre-welding preparation, welding, and post-welding procedures. Figure 1a elaborates on the preparatory steps before welding, including material preparation, equipment installation, and sensor connections. Initially, components, such as flanged axles, were manufactured with various diameters according to the standards established in GB/T 711-2017, where the front half of the flanged axle was machined to create four flat surfaces for subsequent testing. Following this, the welding apparatus was installed to ensure stability during the welding process. The I-beam support base was anchored to the floor, and the flanged plate was fixed to the support surface via spot welding. The flanged axle was also secured to the center of the flanged plate through spot welding. Lastly, sensors were installed; three K-type thermocouples were arranged at different positions along the side surface of the flanged axle and secured with high-temperature ceramic tape to record the thermal cycle curve at the measurement points during welding. In addition, displacement gauges were arranged in three different directions at the end of the flange shaft to record the deformation of the shaft end during welding. Figure 1b illustrates the various welding parameters and conditions utilized in the experiments, including variations in diameter and welding time, emphasizing the authenticity of the experimentation. Finally, Figure 1c depicts the post-welding treatment process for the welded components. To obtain a true representation of the weld pool morphology, the welded component was sliced using a wire-cutting machine and the cross-section was polished with a metallographic grinding and polishing machine. Subsequently, specialized metal etching solution was employed for acid washing of the weld cross-section to facilitate clear observation of the weld pool morphology.

### 2.2. Welding Test

The test specimens were joined by manual arc welding, using a welding machine with adjustable parameters such as voltage and current. The thermal input efficiency of the welding machine was 0.8, with a welding power of 4080 W, a current of 170 A, and a voltage of 24 V. The parameter settings were consistent with the simulation results, and the welding process parameters are listed in Table 2.

**Figure 2:**
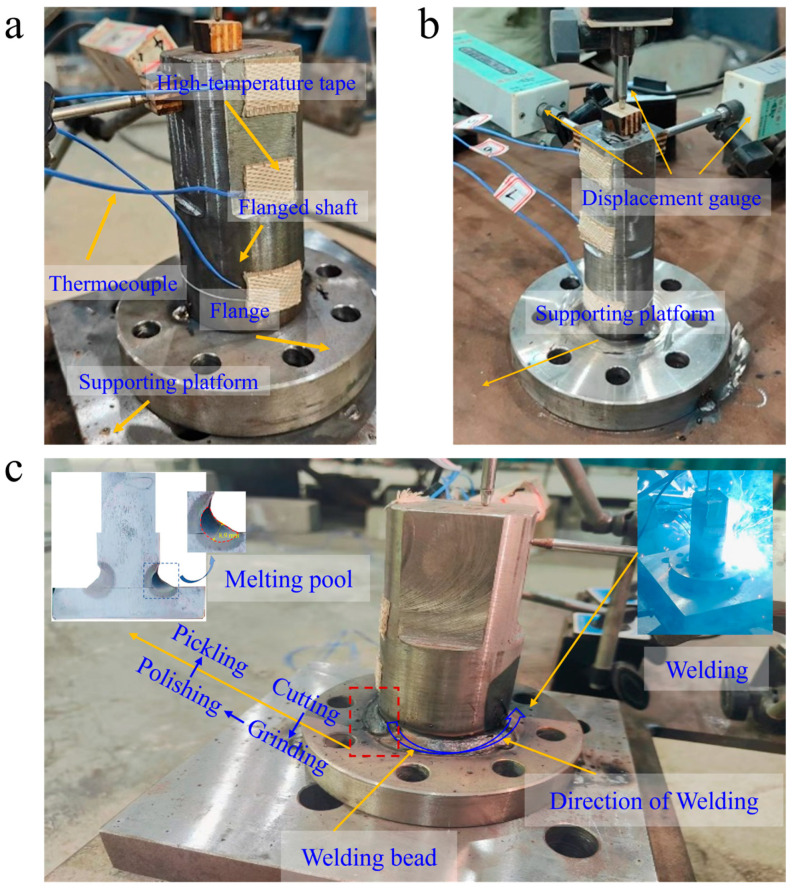
On-site configuration and welding experiment: (**a**) thermocouple locations; (**b**) displacement gauge locations; (**c**) welding process.

Three sets of welding conditions, representing different welding speeds, were established, allowing completion times for welding of 72 s, 90 s, and 180 s, with each condition repeated three times. The on-site setup is illustrated in Figure 2. During the welding process, due to the limitations of the thermocouples’ maximum operational temperature range (−50 °C to 200 °C), it was not possible to monitor the temperature history within the fusion zone (FZ). Thus, as shown in Figure 2a, K-type thermocouples were placed at three positions, 20 mm, 65 mm, and 110 mm from the welding center along the side surface of the flanged axle. The thermocouples were also secured using high-temperature ceramic tape, and the JK3000 multi-channel data logger was used to record the temperature data of characteristic points in real-time during the welding process, generating thermal cycle curves. The measurement accuracy of this data logger is as follows: within the range of −200~1800 °C, the error is ±(reading value × 0.5% + 1) °C, with a display resolution of 0.1 °C. Figure 2b shows the arrangement of displacement gauges (YHD-10 (measuring range: 0–10 mm, accuracy: 0.1%), Liyang Instrument Factory, Changzhou, China). During the welding, the uT8508 dynamic and static electronic strain gauge (produced by Wuhan Youtai Electronic Technology Co., Ltd., Wuhan, China) was used to collect parameters, such as the surface deformation of the shaft end. Meanwhile, displacement gauges were arranged in three different directions at the end of the flange shaft to measure the deformations in the *x*, *y*, and *z* directions, respectively, and insulating wood blocks were used to isolate the current to prevent impacts on the readings of the displacement gauges. Following the completion of welding, the welded components were allowed to cool to near room temperature before disassembling the experimental apparatus. Following GB/T 228.1-2010 standards [41], a CNC wire-cutting machine (DL7750, Taizhou Dongqi CNC Equipment Co., Ltd., Taizhou, China) was used to cut the flanged axle discs vertically along the centerline. After the cutting, the surfaces of the weld pools were polished with sandpaper and a metallographic grinding and polishing machine (MoPao@2B model, Shenzhen Runxing Optical Instrument Co., Ltd., Shenzhen, China). A 4% nitric acid–alcohol metal etching solution (HNO_4_-C_2_H_6_O = 4%) was applied to the cut surface to clean and etch, preparing for observation of the weld pool’s depth and shape, as depicted in Figure 2c, which captures the state of the site following welding. The observable morphology of the weld pool closely resembles a crescent shape.

## 3. Simulation Methodology

Welding is a complex manufacturing process characterized by the interaction of multiple physical phenomena, including the generation and transfer of heat, the flow and solidification of molten metal, the evolution of the material’s microstructure, and the development of welding residual stresses and deformations. Due to this complexity, simultaneously reproducing all the physical processes involved in welding through numerical simulation presents significant challenges [38]. Notably, the thermo-mechanical coupling framework used in calcium sulfoaluminate (CSA) cement hydration-addressing temperature-driven changes in material properties-provided useful insights for this study. It guided our approach to simulating welding residual stresses, as both processes involve material behavior governed by temperature variations [42]. Accordingly, a three-dimensional finite element model was established for the welding of large flanged axles based on thermo-elastoplastic theory. To enhance computational efficiency, uncoupled thermodynamic formulas were employed in the analysis [43,44], allowing for the numerical simulation of temperature fields, deformation fields, and residual stress fields generated during the welding process. A combined heat source model, based on the geometric characteristics of the weld pool, was proposed, ensuring simulation accuracy while reducing the extent of heat source parameter calibration.

### 3.1. Computational Framework

**Figure 3:**
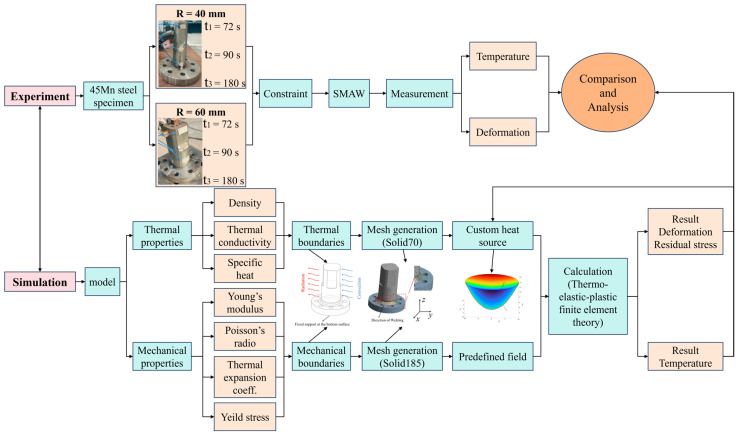
SMAW simulation framework process.

The overall simulation workflow for shielded metal arc welding (SMAW) is illustrated in Figure 3. This includes model establishment, material property definition, thermal and mechanical boundary conditions, meshing, heat source modeling, and simulation of temperature, deformation, and residual stresses using ANSYS 2020 (version: R2) software. A sequential coupling analysis model was utilized in this simulation process. The simulation model was constructed based on experimental conditions and constraints. Historical temperature data and welding deformations were measured during the experiments, enabling a comparative analysis with simulation results to verify the reliability of the numerical model.

### 3.2. Welding Simulation Model

#### 3.2.1. Heat Source Model

Accurate modeling of the heat source is critical for transient temperature field simulations. For processes with significant penetration-such as electron beam welding, laser welding, and GMAW-the planar Gaussian heat source is inadequate. A volumetric heat source model is more appropriate. Given the high computational demand of residual stress analysis, a uniform volumetric heat source offers an efficient compromise and has been validated in numerous studies [45]. Yaghi et al. [46] suggested using 1/10 to 1/5 of the total weld volume as the initial heat source volume, refined through experimental calibration. The thermal flux density *q* within the effective volume is defined as follows:

(1) $$q=\frac{\eta UI}{V}$$

where *q* represents the thermal flux density; η denotes the welding thermal efficiency; *U* signifies welding voltage; *I* indicates welding current; and *V* is the volume of the heat source.

**Figure 4:**
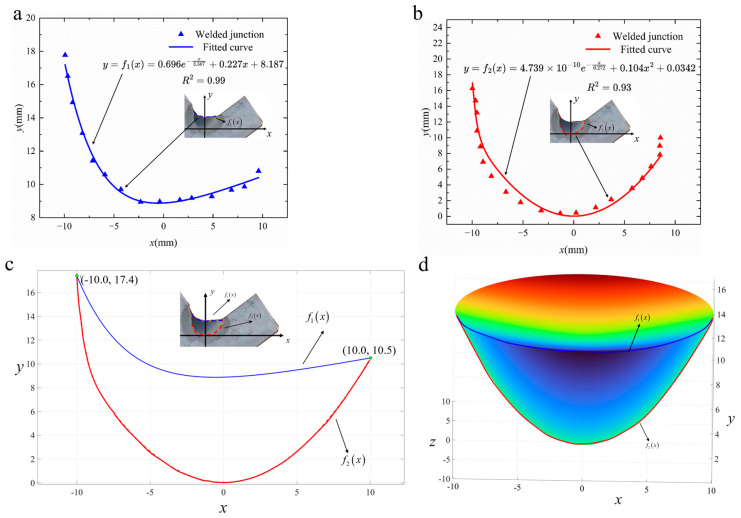
Fitting of actual weld fusion line and spatial distribution of combined heat source model: (**a**) fitted curve of *f*_1_(*x*); (**b**) fitted curve of *f*_2_(*x*); (**c**) combined graph of *f*_1_(*x*) and *f*_2_(*x*); (**d**) schematic diagram of combined heat source model. Curves of different colors represent different contours of the weld: blue stands for the outer contour, red for the inner contour; solid lines represent the fitted curves, and dashed lines represent the extracted contour points.

In this study, a combined heat source model was constructed by fitting the weld pool’s cross-sectional geometry using Origin 2024 software (version SR1). A rotated coordinate system (45° clockwise) was established using the lowest point of the profile as the origin. The fusion line coordinates were extracted using image processing software GetData Graph Digitizer 2.24, and nonlinear fitting produced the equations shown in Figure 4a,b.

The fitted equations, i.e., *f*_1_(*x*) and *f*_2_(*x*), show good fitting with the actual weld fusion line, with *R*^2^ of 0.99, 0.93. The weld pool profile fitting involves some uncertainty, mainly from two systematic errors: manual feature point selection, which introduces positioning deviations, and edge blurring after etching, which adds subjectivity in the defining boundaries. These deviations remain within acceptable limits for welding simulations and do not notably affect the rotational model geometry or simulation reliability.

The next step is to construct a rotational body using boundaries defined by the fitting equations, as depicted in Figure 4c, where the *y*-axis serves as the rotational axis, yielding the rotational body illustrated in Figure 4d. This rotational space defines the region of thermal flux distribution from the molten pool.

The most common distribution model for the heat source is Gaussian distribution, based on experimental observations of heat distribution in arc welding. Assuming the heat is uniformly distributed within the combined heat source model, the thermal flux density at any point within this body equals Q_m_*/V*. *V* is the volume of the combined heat source, which can be calculated as follows:

(2) $$V=2\pi \int_{0}^{x}x\left[f_{2}\left(x\right)-f_{1}\left(x\right)\right]dx$$

where Q_m_ denotes the thermal input from the molten pool, $Q_{m}{=\rho \pi r}_{w}^{2}{\omega H}_{d}$; ρ is the filler wire density; r_w_ represents the filler wire radius, r_w_ = 4.0 mm; ω indicates the wire feed speed, ω = 2.0 m/min; and H_d_ signifies the enthalpy of melting of the filler wire, H_d_ = 270 kJ/kg [40].

The heat introduced by the arc can be represented using a Gaussian surface heat source, where the thermal flux density follows a Gaussian distribution within a circular area of radius r. The arc input power is determined by subtracting the filler wire melting power from the total thermal input, i.e., $Q_{a}=\;Q-Q_{m}$. Thus, the final expression for the combined heat source thermal flux density distribution is given as follows:

(3) $$q\left(x,y,z,t\right)=\left\{\begin{array}{l} \frac{Q_{m}}{V},f_{1}\left(x\right)\leq y<f_{2}\left(x\right)\\ \frac{3Q_{a}}{\pi r^{2}}\cdot e^{-\frac{3\left(x^{2}+z^{2}\right)}{r^{2}}},y=f_{2}\left(x\right) \end{array}\right.$$

For comparison, the thermal flux density distribution of the double-ellipsoidal heat source is expressed as follows:

(4) $$\left\{\begin{array}{l} q_{f}\left(x,y,z\right)=\frac{6\sqrt{3}Qf_{f}}{a_{1}bc\pi \sqrt{\pi }}\exp\left\{-3\left(\frac{x^{2}}{a_{1}^{2}}+\frac{y^{2}}{b^{2}}+\frac{z^{2}}{c^{2}}\right)\right\},x>0\\ q_{r}\left(x,y,z\right)=\frac{6\sqrt{3}Qf_{r}}{a_{2}bc\pi \sqrt{\pi }}\exp\left\{-3\left(\frac{x^{2}}{a_{2}^{2}}+\frac{y^{2}}{b^{2}}+\frac{z^{2}}{c^{2}}\right)\right\},x\leq 0 \end{array}\right.$$

where *f_f_* and *f_r_* represent the energy distribution fractions for the front and rear halves of the heat source, respectively, where the condition *f_f_* + *f_r_* = 2. The length of the front and rear halves of the ellipsoid are denoted as *a_1_* and *a_2_*, respectively, while *b* and *c* represent the width and depth of the ellipsoid.

#### 3.2.2. Thermal Conductivity Calculation

To simplify the calculations, the effects of fluid flow have been neglected. To illustrate the influence of fluid flow on the temperature field of the welding pool, an artificial enhancement of thermal conductivity was employed when temperatures exceeded the melting point of the base material [47]. The transient temperature field during the welding process is calculated according to Fourier’s law [48]:

(5) $$\rho \left(T\right)c_{p}\left(T\right)\frac{\partial T}{\partial t}=\frac{\partial }{\partial x}\left(k\left(T\right)\frac{\partial T}{\partial x}\right)+\frac{\partial }{\partial y}\left(k\left(T\right)\frac{\partial T}{\partial y}\right)+\frac{\partial }{\partial z}\left(k\left(T\right)\frac{\partial T}{\partial z}\right)+Q_{v}$$

where *T* represents the temperature; *c_p_*, *k*(*T*) and *ρ*(*T*) respectively define the specific heat, thermal conductivity, and density related to temperature; *t* signifies time; and *Qv* indicates the thermal energy density.

#### 3.2.3. Thermal Radiation Calculation

The heat exchange at the surface boundaries of the workpiece typically involves both convective and radiative heat transfer [23]. The boundary conditions, which encompass both convective and radiative heat losses, are described by the following equation:

(6) $$-k\left(T\right)\frac{\partial T}{\partial n}=h_{c}\left(T-T_{0}\right)+{\varepsilon \sigma }_{B}\left(T^{4}-T_{0}^{4}\right)$$

where h_c_ denotes the heat transfer coefficient between the structure and the surrounding environment, h_c_ = 25 W/(m^2^·°C); ε represents the emissivity of the surface, ε = 0.8; σ_B_ is the Stefan–Boltzmann constant, σ_B_ = 5.670 × 10^−8^ W/(m^2^·°C^4^); and *T* and T_0_ represent the temperature of the workpiece and the initial temperature of the surrounding environment, respectively.

#### 3.2.4. Mesh Model and Boundary Conditions

During the simulation of welding, the weld region experiences highly concentrated thermal flux input that gradually progresses along the weld-a characteristics that necessitates the careful selection of element mesh types [49,50]. Specifically, the temperature field simulations employ solid eight-node hexahedral elements (Solid70) for thermal analysis. To transfer temperature field data to the structural model, the stress field simulations utilize solid eight-node hexahedral elements (Solid185), which can automatically convert from Solid70 elements during the thermal–mechanical coupling process [51,52,53].

To assess the mesh sensitivity, this study implemented a series of mesh schemes with varying element sizes. The refined region (e.g., the weld zone and nearby areas with steep stress and temperature gradients) used mesh sizes from 0.5 mm to 2 mm, while the non-refined region (areas with smoother field variations) ranged from 1 mm to 4 mm, as shown in Table 3. By comparing the peak temperatures and the times to reach peak temperatures of measuring points under different mesh sizes, it was found that when the mesh size in the refined area was 1 mm and that in the non-refined area was 2 mm, further refining the mesh size had almost no impact on the accuracy of the simulation results but would lead to slow calculation efficiency due to a sharp increase in the number of meshes. Therefore, the mesh for the weld area and its adjacent heat-affected zone were refined, with a mesh size of 1 mm in the refined area and 2 mm in the non-refined area. This approach ensures both the efficiency and accuracy of the simulated temperature and stress fields.

**Figure 5:**
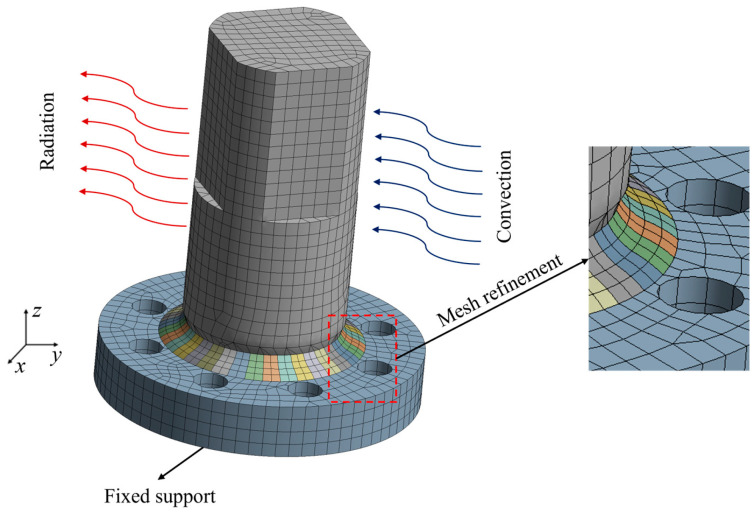
Mesh model and boundary conditions.

In the mechanical analysis, constraints are applied to prevent the rigid body motion of the workpiece. Although the details of the weld cross-section morphology may vary significantly in experiments, their effect on the temperature and stress fields at the microstructural level is minimal. Therefore, variations in the weld morphology are disregarded, and simulations are based exclusively on an idealized cross-sectional geometry, as illustrated in Figure 5.

Mechanical boundary conditions must be applied during the stress field calculations to prevent the absence of necessary constraints, which could result in rigid body displacement under the load. The boundary conditions for the model closely reflect actual conditions: the thermal analysis boundary conditions include both convective and radiative heat transfer, while the structural analysis boundary conditions impose fixed constraints in all three directional displacements on the bottom surface of the model, as shown in Figure 5.

In addition, in the finite element simulation of this study, the implicit algorithm was adopted. For the welding phase, a small time step of 0.001 s was set to accurately capture key details such as the weld pool formation and rapid temperature rise, while the time step was adjusted to 0.01 s for the cooling phase to improve computational efficiency while ensuring the accuracy of the stress evolution law.

### 3.3. Finite Element Analysis of Welding Strain Field

In finite element analysis, the total strain at each material point can be represented by the following equation:

(7) $$\varepsilon_{\mathrm{total}}{=\varepsilon }_{\mathrm{elastic}}{+\varepsilon }_{\mathrm{plastic}}{+\varepsilon }_{\mathrm{thermal}}{+\varepsilon }_{\mathrm{creep}}{+\varepsilon }_{\mathrm{phase}}$$

where ε_total_ is the total strain at each material point and ε_elastic_, ε_plastic_, ε_thermal_, ε_creep_ and ε_phase_ signify the strains due to elastic deformation, plastic deformation, thermal effects, creep, and phase change, respectively. Considering the short duration and high temperature of the welding process, the effect of creep on the structure’s macroscale residual stresses and deformations is minimal and can thus be neglected in this study [54]. Similarly, the phase change-induced strains are not considered. Consequently, the total strain at each material point can be simplified as follows:

(8) $$\varepsilon_{\mathrm{total}}{=\varepsilon }_{\mathrm{elastic}}{+\varepsilon }_{\mathrm{plastic}}{+\varepsilon }_{\mathrm{thermal}}$$

**Figure 6:**
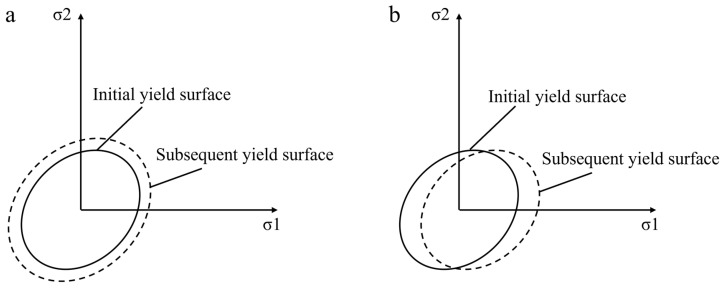
Hardening criterion model: (**a**) isotropic hardening model; (**b**) kinematic hardening model.

For metal materials, the stress–strain dependence shows linear elasticity below the proportional limit (Hooke’s law) and nonlinear plasticity above the yield limit, where the stress state depends on the strain history. Welding involves material nonlinearity (plasticity) and geometric nonlinearity, so the plastic behavior is calculated by the incremental theory (yield, flow, and hardening criteria, as shown in Figure 6). The commonly used yield criterion is the Von Mises yield criterion. When the equivalent stress exceeds the yield stress of the material, plastic deformation will occur. In the three-dimensional principal stress space, the equivalent stress is defined as follows:

(9) $$\bar{\sigma }=\frac{\sqrt{2}}{2}\sqrt{{\left(\sigma_{1}-\sigma_{2}\right)}^{2}+{\left(\sigma_{2}-\sigma_{3}\right)}^{2}+{\left(\sigma_{3}-\sigma_{1}\right)}^{2}}\leq \sigma_{s}$$

where $\bar{\sigma }$ is the equivalent stress and $\sigma_{1}$, $\sigma_{2}$, $\sigma_{3}$ denote the normal stresses acting along three directions that are orthogonal to each other, respectively. $\sigma_{s}$ is the uniaxial tensile yield limit of the material.

Once the material yields, plastic flow occurs. The flow criteria of plastic strain increment and stress state are as follows:

(10) $${\left\{d\varepsilon \right\}}_{p}=d\lambda \frac{\partial \bar{\sigma }}{\partial \left\{\sigma \right\}}$$

where ${\left\{d\varepsilon \right\}}_{p}$ is the plastic strain increment; dλ is the plastic multiplier; and $\frac{\partial \bar{\sigma }}{\partial \left\{\sigma \right\}}$ is the partial derivative of the scalar function $\bar{\sigma }$ with respect to the vector function $\left\{\sigma \right\}$.

For metal materials, the hardening criteria are commonly used in the above two; however, in the actual numerical simulation, the results of welding residual stress are very different when different hardening criteria are selected, as shown in Figure 6. Zhang et al. [55] studied the influence of different hardening criteria on welding residual stress. The results of the kinematic hardening criterion are more accurate. Therefore, for the numerical simulation in this paper, we adopt the kinematic hardening criterion.

Thermal strains, induced by temperature changes, are related to material thermal expansion coefficients and can be expressed using the following equation [56]:

(11) $$\varepsilon_{\mathrm{thermal}}=k\cdot \Delta T$$

Additionally, to balance the computational accuracy with efficiency, the phase changes and re-melting of materials have been disregarded.

### 3.4. Material Proporties

45Mn steel is known for its excellent comprehensive performance as a high-quality carbon structural steel. However, research on the welding simulation of this material, particularly regarding its high-temperature properties, is relatively scarce, resulting in a lack of systematic support for its attribute parameters. In finite element analysis, it is often suitable to simplify parameters that have a minimal impact on the results. For instance, neglecting high-temperature structural phase changes in thermal stress calculations can enhance modeling efficiency [57]. The experimental determination of thermal–physical and mechanical properties is often time-consuming, costly, and complex [38]. To improve efficiency, in this study we employed thermodynamic simulations of the heat treatment process of 45Mn steel using JMatPro 7.0.0 software [58,59,60], allowing the extraction of key thermal–physical parameters for use in numerical analysis and offering valuable reference data for subsequent modeling and engineering applications.

Various factors, including temperature, grain size, alloying elements, and microstructural evolution, were considered in the systematic calculation of thermal–physical parameters for both 45Mn steel and E5015 filler material over the temperature range of 25 °C to 1600 °C. The results are summarized in Table 4.

## 4. Results and Discussion

### 4.1. Validation of the Welding Heat Source Model

In sequentially coupled thermodynamic analyses, the accuracy of the thermal field directly affects the reliability of subsequent mechanical analyses. The validity of the simulated temperature field is strongly correlated with the degree to which the shape and size of the weld pool align with experimental or theoretical expectations. To enhance the fidelity of the simulation, this study validates the welding heat source model by comparing the simulated and experimental weld pool geometry and thermal cycle curves.

**Figure 7:**
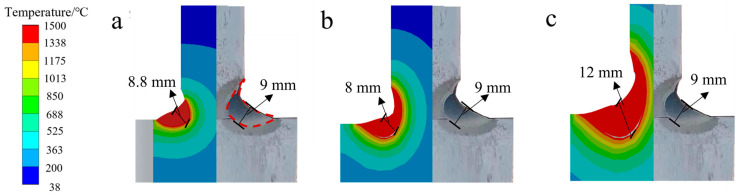
Comparison of the geometric shape and dimensions of the weld crosssection’s molten pool: (**a**) combined heat source model; (**b**) uniform volumetric heat source model; (**c**) double-ellipsoidal heat source model.

Based on the simulated temperature field results, the weld pool geometries, produced by three different heat source models, were compared to the experimental results, as shown in Figure 7. According to the literature [61], the melting point of 45Mn steel is 1497 °C. The combined heat source model, constructed using the actual geometric profile of the molten pool, showed excellent agreement with experimental observations. The simulated weld pool depth was 8.8 mm, just 2.2% less than the measured depth of 9 mm. This accuracy is achieved without additional calibration of shape parameters, thanks to the inclusion of real fusion line data. In contrast, the homogeneous volumetric and double-ellipsoidal models exhibited significant deviations, with errors of 11.1% and 33.3%, respectively. These results confirm that the combined heat source model provides a more realistic representation of the actual weld pool geometry.

**Figure 8:**
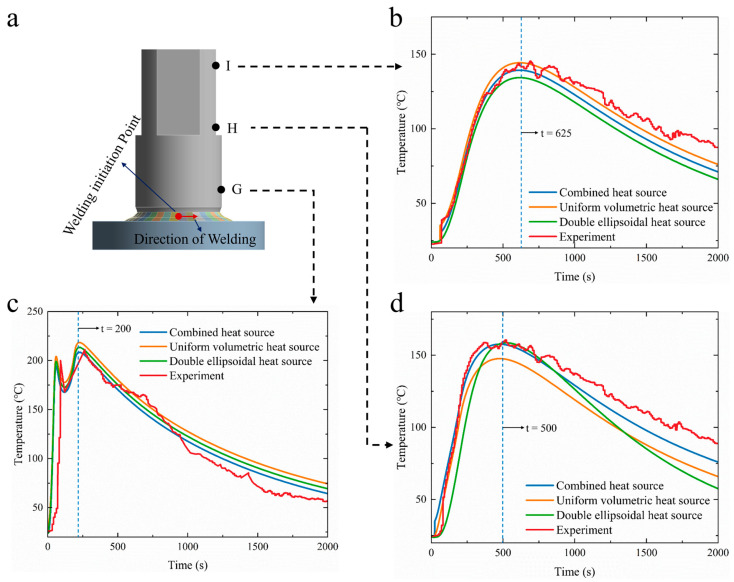
Comparison of thermal cycle curves (welding time: 180 s): (**a**) measurement point locations; (**b**) thermal cycle curve at point I; (**c**) thermal cycle curve at point G; (**d**) thermal cycle curve at point H.

To further validate the heat source model, a flange shaft of a diameter of 60 mm and a welding time of 180 s were simulated in a finite element analysis, comparing the generated thermal cycle curves with experimental results. The positioning of K-type thermocouples and the resultant thermal cycle curves are illustrated in Figure 8. Analyzing the thermal cycle curves at points G, H, and I—perpendicular to the welding direction—it is noted that the thermal cycle curve obtained from the combined heat source model follows the same trend as those from the other two models. However, regarding the peak values, the results from the combined heat source model exhibit a closer alignment with experimental values. As the distance from the weld seam increases, the time taken to reach peak temperatures at points G, H, and I is delayed, with peak temperatures subsequently decreasing. To quantitatively assess the accuracy of the simulation, the root mean square error (RMSE) and the maximum deviation between the simulated and experimental thermal cycles were calculated at three representative measurement points (G, H, and I). Among the three heat source models evaluated, the combined heat source model demonstrated the best agreement with the experimental data, yielding an overall RMSE of 4.2 °C and a maximum deviation of 8.3 °C, which was observed during the cooling phase at point G. In contrast, the uniform volumetric and double-ellipsoidal models resulted in higher RMSE values of 7.6 °C and 6.1 °C, and maximum deviations of 15.2 °C and 11.5 °C, respectively. These quantitative results indicate that the combined model not only captures the peak temperatures more accurately but also maintains better consistency throughout the entire thermal cycle. This confirms its superior capability in simulating both the transient thermal response and the spatial distribution of heat during the welding process, effectively overcoming the limitations of localized curve fitting observed in the other two models.

As shown in Figure 8, for the thermal cycle curve at point I, located furthest from the weld seam, the time to reach peak temperature was 625 s, with the experimental peak temperature measuring 145 °C and the combined heat source model predicting peak temperatures of 142 °C, resulting in an error of only 2.1%. In comparison, the other two models yielded peak temperatures of 128 °C and 148 °C, with errors of 11.7% and 2.1%, respectively. At point G, closest to the weld seam, the peak temperature was attained after 200 s during the experiment, measuring 210 °C, while the combined heat source model yielded a peak temperature of 206 °C, demonstrating a minimal error of 1.9%. For point H, the thermal cycle curve closely mirrored the experimental result, with a peak temperature of 160 °C observed experimentally versus 157 °C in simulation, yielding an error of just 1.9%, as shown in Table 5.

However, it was noted that the cooling rates during the welding phase at points H and I differed, as recorded experimental cooling rates were lower than those predicted by the model, as shown in Figure 8b,d. This discrepancy is attributed to variations in the thermal convection boundary conditions between the actual and simulated scenarios. During the welding experiment, the airflow around the components was complex and unstable, leading to dynamic fluctuations in the convective heat transfer coefficient. Conversely, in the simulation, a constant convective heat exchange was assumed between the air and the components, which is an idealization that does not account for the non-stationary nature of actual conditions. This mismatch in theoretical boundary conditions and real-world thermal dynamics led to discrepancies between simulated and experimental cooling rates. Overall, the findings indicate that the combined heat source model based on molten pool geometric features provides a more accurate simulation of actual welding conditions, further validating the model’s accuracy.

**Figure 9:**
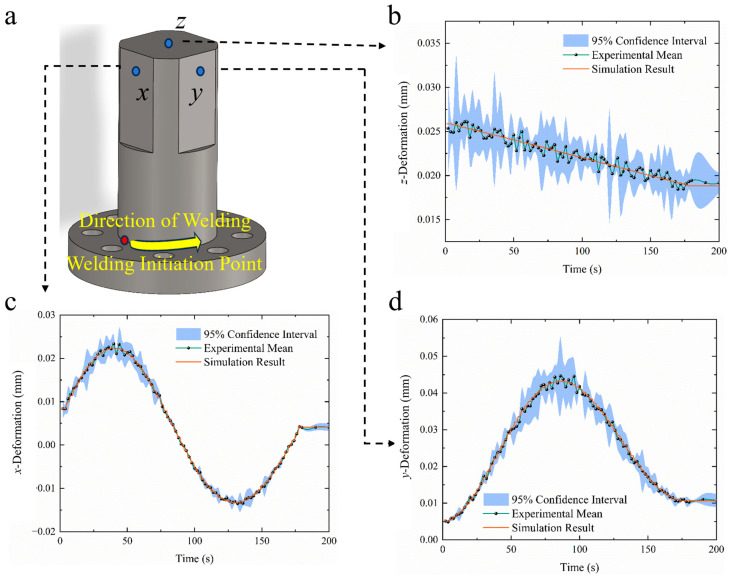
Comparison of experimental and simulated deformation (welding time: 180 s): (**a**) measurement point locations; (**b**) *z* direction; (**c**) *x* direction; (**d**) *x* direction.

In addition to the thermal cycle validation, deformation data at key measuring points, such as *x*, *y* and *z*, were used to further evaluate the accuracy of the heat source models. To account for experimental variability, a 95% confidence interval (CI) was constructed based on three repeated measurements, as shown in Figure 9.

The simulated deformations in the *x*, *y*, and *z* directions were compared against the mean experimental measurements at key reference points. The results demonstrated that the combined heat source model provided the highest level of agreement, with nearly all predicted displacements falling within the 95% confidence intervals derived from repeated experimental trials. Notably, the width of the 95% confidence interval quantitatively reflects experimental uncertainty. As shown in Figure 9, larger variations observed in the *z* direction suggest greater sensitivity to environmental disturbances, whereas the narrower and more stable intervals in the *x* and *y* directions indicate stronger deformation regularity and improved model predictability. These results indicate that the combined heat resource model not only captures the thermal evolution more accurately, but reliably predicts the structural deformation induced by welding.

### 4.2. Finite Element Analysis of Welding

In welding processes, temperature, deformation, and residual stress fields exhibit intrinsic interdependencies: thermal gradients drive deformation through differential thermal expansion, while constrained deformation, in turn, dictates the distribution of residual stresses. Understanding these coupled dynamics is critical to unraveling the underlying thermo-mechanical behavior.

#### 4.2.1. Analysis of the Welding Temperature Field

**Figure 10:**
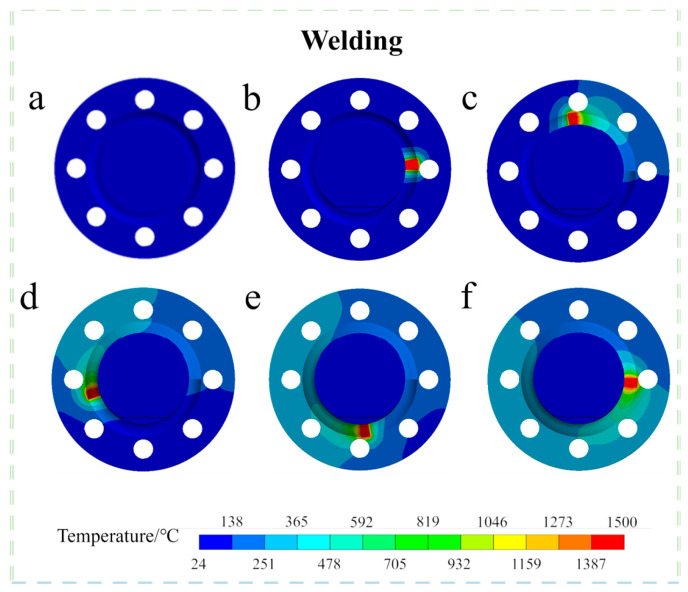

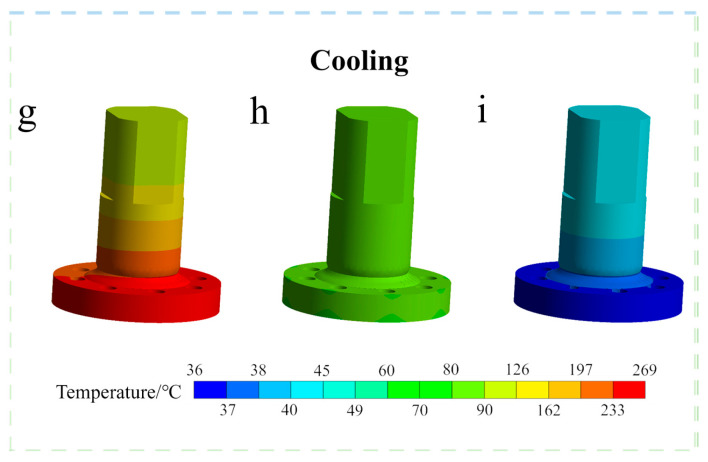
Temperature field at various moments during the welding process (welding time: 180 s): (**a**) t = 0 s; (**b**) t = 5 s; (**c**) t = 50 s; (**d**) t = 100 s; (**e**) t = 140 s; (**f**) t = 180 s; (**g**) t = 300 s; (**h**) t = 1500 s; (**i**) t = 3180 s.

To study the variations in the temperature field during the arc welding of large flange shafts, finite element models of a diameter of 60 mm and a welding time of 180 s were selected for analysis. Figure 10 illustrates the temperature field distribution cloud of the welded 45Mn steel flange shaft over time during the arc welding process. Temperature field results were analyzed at the following moments: 0 s, 5 s, 50 s, 100 s, 140 s, 180 s, 300 s, 1500 s, and 3180 s. Here, 0 s marks the start of welding, while 180 s marks its completion, with 5 s to 140 s representing the intermediate stage and 300 s to 3180 s representing the cooling phase.

The data presented in Figure 10 show that the temperature of the workpiece changes rapidly at the onset of welding. In Figure 10b, the heat source moves, causing the temperature to rise from 24 °C to 1500 °C within 5 s. At the 50 s mark, the maximum temperature is maintained at 1500 °C, transitioning into a relatively stable phase that maintains this maximum temperature until welding concludes. Once the welding is completed and cooling commences, the workpiece temperature begins to decline, falling below 40 °C by 3180 s, marking the end of the entire welding process. Due to the varying thermophysical properties of the material at different temperatures, cooling occurs non-uniformly throughout all areas during the welding process. During the application of the combined heat source, the temperature diffusion forms a more regular pattern. As cooling progresses, this area expands while the temperature decreases. An analysis of the rate of temperature decrease reveals that, while the workpiece temperature is initially high, the temperature drops rapidly. However, as the workpiece cools, the cooling rate gradually slows, further verifying the reliability of the simulation results.

**Figure 11:**
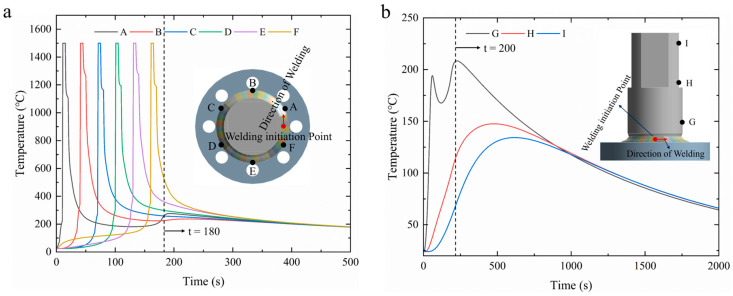
Temperature variation in a 60 mm diameter workpiece during welding: (**a**) thermal cycle curves along the welding direction (points A–F); (**b**) thermal cycle curves along the thickness direction (points G–I).

During the welding process, two reference paths were established to analyze temperature changes at various locations on the workpiece. One path was along the welding direction, while the other traveled across the thickness of the component. Six surface points (A–F) were selected at equal angles and distances from the welding start point along the welding direction, spaced 60° apart. Three surface points (G–I) were taken along the thickness direction, with a distance of 45 mm between points, transitioning from close to the weld seam to further away, as depicted in Figure 11.

Figure 11a,b show the thermal cycle curves at points along the two different paths. It is evident that along the welding direction, the thermal cycle curves for each point exhibit a consistent overall trend, with variations limited to the timing of peak temperatures. As the welding heat source approaches and reaches each sampling point, the temperatures increase at a rapid rate, reaching a peak temperature of 1500 °C. As the heat source moves away, the temperatures at the sampling points gradually decrease. For each sampling point, peak temperatures are reached several seconds after welding concludes. As the moving heat source encounters the sampling line in the vertical welding direction, the temperature at each point along the thickness direction experiences a rapid increase, with the point closest to the weld, point G, reaching a peak temperature of 206 °C within 20 s after welding completion. Points H and I reached their peak temperatures at 500 s and 625 s, respectively. Additionally, as the distance from the weld center increases, peak temperatures decrease sharply, resulting in significant temperature gradients. By 2000 s, the temperature difference across all the nodes becomes minimal, indicating that the temperature distribution within the workpiece has stabilized.

#### 4.2.2. Deformation Field Analysis

**Figure 12:**
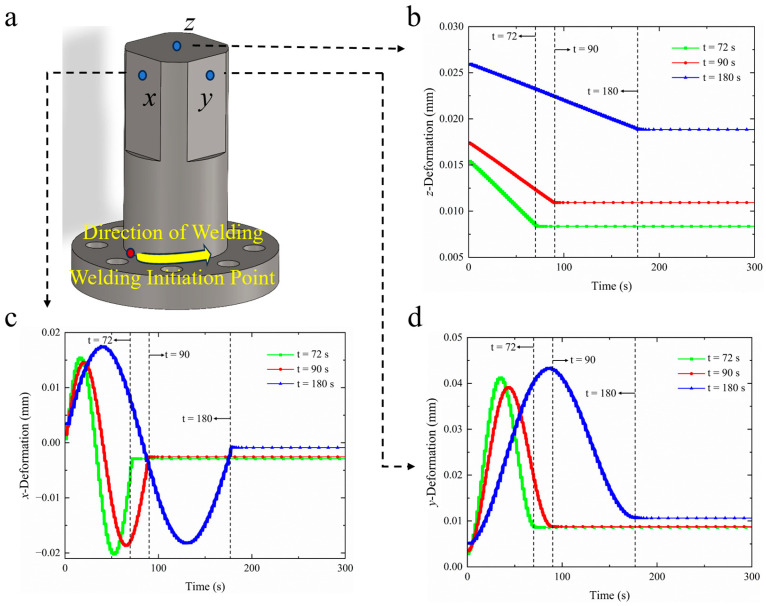
Measurement points and deformation curves from the finite element simulations: (**a**) measurement point locations; (**b**) *z* direction; (**c**) *x* direction; (**d**) *y* direction.

To further demonstrate the efficacy of the heat source model, three different welding time simulations of a flange shaft of a diameter of 60 mm were conducted, with measurement locations as depicted in Figure 12a. The coordinate system is consistent with the global coordinate system, with its origin located at the center of the flange base. The *x*-axis aligns with the line connecting the center of the flange base to the welding starting point, the *z*-axis corresponds to the central axis of the flange, and the *y*-axis is perpendicular to the xOz plane. In the figure, the *x* and *y* directions represent positions 10 mm from the shaft end, while the *z* direction corresponds to the center of the shaft end plane. Figure 12b–d present the deformation curves in the *z*, *x*, and *y* directions for welding times of 72 s, 90 s, and 180 s, respectively. Upon observing the deformation curves, it is apparent that the deformation behavior during the welding does not vary significantly with changes in welding speed or duration; rather, the peak deformation values and the times achieved differ. With longer welding durations, the time to reach peak deformation in the flange shaft extends. Figure 12c illustrates the deformation curve in the *x* direction. Throughout the entire welding process, under all three welding durations, the deformation of the flange shaft in the *x* direction initially increases, decreases, and then increases again, finally stabilizing at around 0.003 mm after cooling completion. Figure 12d shows the deformation curve in the *y* direction; with ongoing welding, the deformation in the *y* direction increases, peaking at approximately 0.04 mm halfway through the weld. As welding continues, the deformation in the *y* direction starts to decline, as measurement point *y* is positioned at the front half of the welding segment. Once welding transitions to the latter half, the *y* direction’s deformation typically increases again due to the welding process, causing a reduction in the deformation at measurement point *y*. Finally, after welding is complete, the deformation stabilizes at approximately 0.01 mm. Figure 12b presents the deformation curve in the *z* direction, where the deformation in the *z* direction of the flange shaft slowly decreases as welding progresses, eventually reaching a peak deformation value at the end of the welding process. Comparing the deformation curves across the three welding durations reveals that, at a duration of 90 s, the flange shaft exhibits a relatively low *z* direction deformation of 0.0070 mm, while at 180 s, the deformation from start to finish is 0.0063 mm but the peak deformation is maximum at 0.0258 mm. A 72 s duration results in a peak deformation of only 0.015 mm but a total deformation of 0.0075 mm. Consequently, during a welding duration of 90 s, it is easier to predict the deformation of the workpiece and implement appropriate measures in a timely manner.

**Figure 13:**
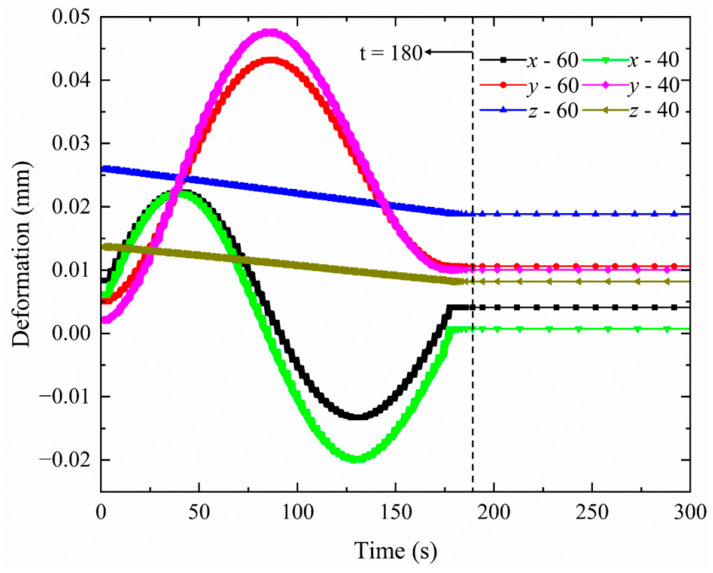
Comparison of deformation curves for different diameters (60 mm and 40 mm).

Figure 13 presents the deformation curves for flange shafts of diameters of 60 mm and 40 mm under a welding duration of 180 s. Comparing the deformation curves, it is evident that with identical welding durations, the deformation behavior of both workpieces remains consistent, though their peak values differ. Examining the deformation curve for the flange shaft in the *x* direction, it is observed that throughout the welding process, the 40 mm diameter shaft consistently exhibits greater deformation than the 60 mm diameter shaft. The deformation curve for the *y* direction illustrates that as welding progresses, the deformation continues to increase, with the maximum peak deformation for the 40 mm diameter shaft exceeding that of the 60 mm diameter shaft. After continued welding, *y* direction deformation begins to diminish. Ultimately, at the conclusion of welding, the deformation stabilizes. An analysis of the deformation curve in the *z* direction demonstrates that as welding progresses, the deformation of the flange shaft in the *z* direction slowly decreases. Overall, it can be concluded that with other conditions held constant, an increase in the workpiece diameter enhances the overall structural stiffness, permitting the stiffer components to better resist the deformation induced by thermal input. Moreover, as the diameter increases, the proportion of the weld relative to the total cross-section of the workpiece diminishes, reducing the size of the heat-affected zone relative to the whole and lessening the deformation resulting from uneven thermal input.

#### 4.2.3. Analysis of Welding Residual Stress Field

**Figure 14:**
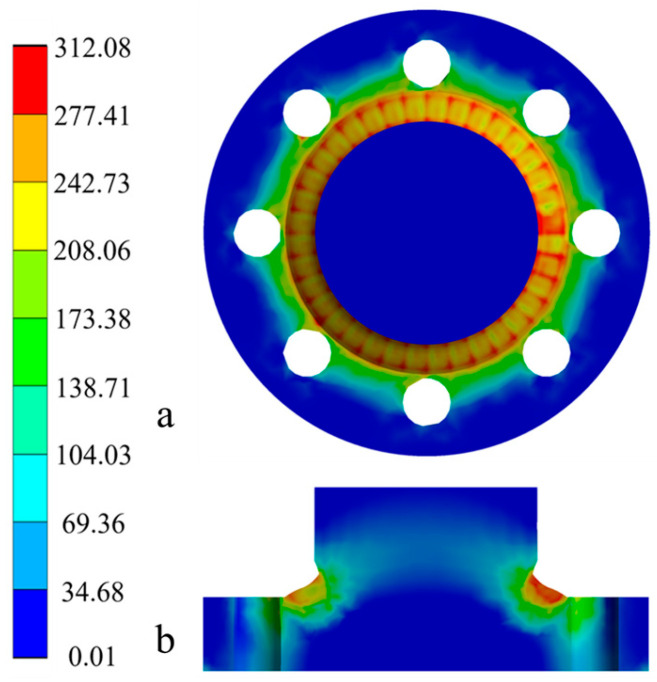
Equivalent residual stress distribution cloud diagram for flange shaft welding: (**a**) top view; (**b**) cross-sectional view.

After the welding, as the welded component gradually cools to room temperature, the stress field distribution stabilizes, with the residual stress distributions on the surface and along the central cross-section. Figure 14 illustrates the distribution of equivalent residual stress. A uniform circumferential distribution of overall stress radiates outward from the center of the weldment, primarily due to the isotropic material properties of the base material. The high stresses, with a maximum value of 312 MPa, are predominantly concentrated in the weld seam and its connection area with the base material, where the equivalent residual stress is significantly higher and more concentrated. The stress cloud diagram of the cross-section indicates that the equivalent residual tensile stress is predominantly located in the weld seam and adjacent areas, diminishing from the seam outward, with a significant stress gradient present within the weld seam region.

**Figure 15:**
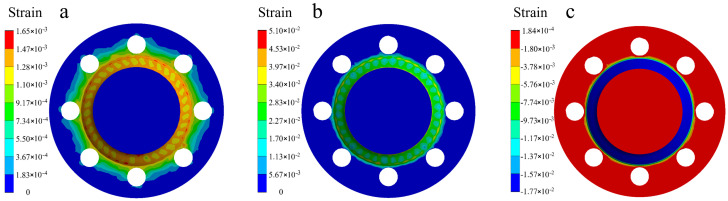
Strain distribution cloud diagram for the flange shaft welding: (**a**) elastic strain; (**b**) plastic strain; (**c**) thermal strain.

The strain distribution cloud diagram for the welded flange shaft is shown in Figure 15, including elastic strain, plastic strain, and thermal strain. It is observable that during the welding, the strain primarily concentrates in the weld seam and near-weld region, predominantly driven by plastic strain, which peaks at 0.051, serving as the main source of residual stress. The maximum thermal strain is −0.0177, while the maximum elastic strain is 0.00165; both of these values are relatively small compared to plastic strain, further confirming that plastic deformation is the dominant factor contributing to the generation of residual stress.

#### 4.2.4. Influence of Welding Time on Residual Stress

**Figure 16:**
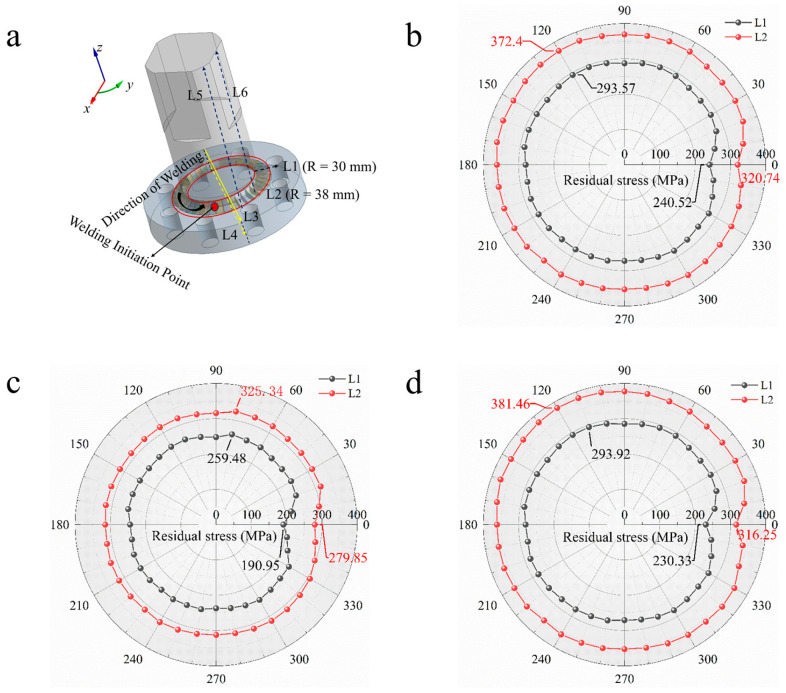
Sampling line distribution for L1,L2 and residual stress distribution curves: (**a**) distribution of residual stress sampling lines; (**b**) residual stress distribution for welding time of 72 s; (**c**) residual stress distribution for welding time of 90 s; (**d**) residual stress distribution for welding time of 180 s.

To facilitate a more comprehensive analysis of the internal residual stress distribution within the workpiece, residual stress data were extracted from multiple locations across the component and compared. Specifically, to investigate the distribution of welding residual stress along the welding direction, sampling lines L1, L2 were taken, where L1 is a circular trajectory line with a radius of 30 mm on the *z* = 20 mm plane, and L2 is a circular trajectory line with a radius of 38 mm. To explore the vertical distribution of residual stress perpendicular to the welding direction, sampling lines L3, L4 were used, with L3 positioned on the *z*= 20 mm plane (the surface of the flange) and L4 on the *z*= 10 mm plane (at half the thickness of the flange). To investigate the distribution of residual stress along the welding thickness direction, sampling lines L5, L6 were employed. The distribution of residual stress sampling lines is illustrated in Figure 16a.

To examine the distribution of welding residual stress along the welding direction, sampling lines L1, L2 were analyzed. Figure 16 presents the residual stress distribution curves for sampling lines L1, L2 over different welding durations. Figure 16a shows the sampling positions for lines L1, L2. Figure 16b–d display the residual stress distribution curves in polar coordinates along the sampling lines L1 and L2 for welding durations of 72 s, 90 s, and 180 s. In this polar coordinate system, angles from 0° to 360° (counterclockwise) represent the welding direction, while the radial distance indicates the magnitude of residual stress. Figure 16b illustrates the residual stress distribution curve for high-speed welding conditions. Notably, the residual stress at the welding start point does not reach maximum levels; this is due to the rapid heating and expansion of the weld area caused by the arc, counteracted by the surrounding cold base material, which induces thermal plastic compression. However, since the constraints at the start position are relatively minor, the stress values do not peak. As welding progresses, the contraction in the area behind the weld is strongly constrained by the already welded section, leading to a gradual increase in residual stress that peaks in the middle of the weld. As shown in Table 6, the residual stress peak for sampling line L1 reaches 294 MPa, while for line L2 it reaches 372 MPa. As welding approaches its completion, a “secondary thermal cycle” occurs, reducing the temperature gradient and achieving equilibrium in the closed weld structure, resulting in a slight reduction in residual stress. Figure 16c,d illustrate the residual stress distribution curves for medium-speed and low-speed welding. From the trends observed in the distribution curves, it was found that they align with those of high-speed welding, with the only difference being evident in the magnitudes of the residual stresses. In the case of a 90 s welding time, the peak residual stress for sampling line L1 measures 259 MPa, while for line L2 it records 325 MPa. For a welding time of 180 s, the peak for line L1 is 294 MPa and for line L2 it is 381 MPa. This comparison suggests that medium-speed welding can alleviate residual stress accumulation, as insufficient heat conduction during high-speed welding leads to steep temperature gradients in the weld and nearby regions, while areas further from the weld remain cooler and exhibit greater stiffness, resulting in enhanced resistance to deformation. Rapid cooling prevents the adequate release of plastic strain through overall coordination, increasing residual stress. Conversely, during slow welding, excessive heat input can lead to localized over-plastic deformation, with extreme temperatures in the weld and surrounding regions extending the duration the material is in a plastic state. Simultaneously, material in the high-temperature zone surrounded by cooler zones experiences compressive plastic deformation, and insufficient strain release during cooling leads to grain coarsening and decreased plastic reserves, exacerbating stress concentration and raising residual stress levels. Furthermore, as observed during welding at 72 s, the difference in residual stress between sampling lines L1 and L2 approached 78 MPa, whereas this difference diminished to approximately 66 MPa at 90 s, indicating that an appropriately extended welding time aids in eliminating gradients of stress perpendicular to the welding direction, thereby reducing structural warping and deformation tendencies. Overall, residual stress distributions were uniformly observed along the welding direction, with the trend being consistent across different welding durations: residual stress gradually increases from welding initiation, peaks as the welding arc stabilizes, and then maintains small fluctuations until the welding process ceases, exhibiting a characteristic trend of “initial increase–stable arc leading to peak maintenance–decrease upon welding completion.”

**Figure 17:**
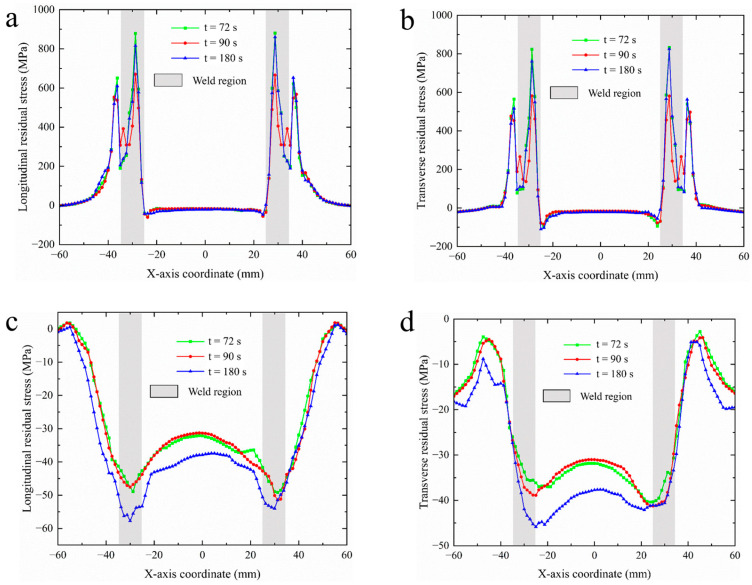
Residual stress distribution curves for sampling lines L3,L4 under varying welding durations: (**a**) longitudinal residual stress for L3; (**b**) transverse residual stress for L3; (**c**) longitudinal residual stress for L4; (**d**) transverse residual stress for L4.

Research into the distribution patterns of residual stress perpendicular to the welding direction was conducted utilizing the residual stress distribution curves from sampling lines L3 and L4, as shown in Figure 17, and the residual stress data was presented in Table 7. Overall, it was observed that for both sampling lines, the peak longitudinal residual stress consistently exceeded the peak transverse residual stress, with similar trends in variation. Figure 17a,b illustrate the longitudinal and transverse residual stress distribution curves for sampling line L3. By comparing the residual stress distribution curves at different welding durations, it was evident that when the welding time was 90 s, both the longitudinal and transverse residual stress peaks were minimized, with longitudinal tensile stress peaking at 659 MPa and transverse tensile stress at 587 MPa. Under the other two welding durations, the longitudinal tensile stress peaks remained closely aligned at 894 MPa, while the transverse tensile stress reached 826 MPa. The residual stresses along sampling line L3 exhibited symmetrical distributions, with residual tensile stress peaks evident in the weld area, wherein longitudinal residual tensile stress exceeded transverse residual tensile stress. Notably, a suitable extension of welding duration effectively reduces residual stress in welded components, with longitudinal residual stress decreasing by 235 MPa (a reduction of 36%) and transverse residual stress decreasing by 239 MPa (a reduction of 41%).

Figure 17c,d provide the longitudinal and transverse residual stress distribution curves for sampling line L4. In contrast to line L3, sampling line L4’s entire path predominantly reflects residual compressive stress, with the peak compressive stress occurring in the weld area. As one moves further from the weld region, the residual compressive stress diminishes, attributed to the higher thermal input in locations closer to the weld, which results in steeper temperature gradients and the surrounding metal expanding due to heat, consequently exerting compressive stress on the material at sampling line L4. The longitudinal residual stress appears as a “W” pattern in Figure 17c; under different welding durations, 180 s yields greater longitudinal compressive stress compared to the other two durations, peaking at 58 MPa. For both longitudinal and transverse residual stresses, fluctuations are more pronounced in the weld area, while stresses in the base material exhibit relative steadiness, indicating that the thermal welding process impacts the weld seam and its vicinity more significantly than the base material, prompting complex stress evolution in these areas.

**Figure 18:**
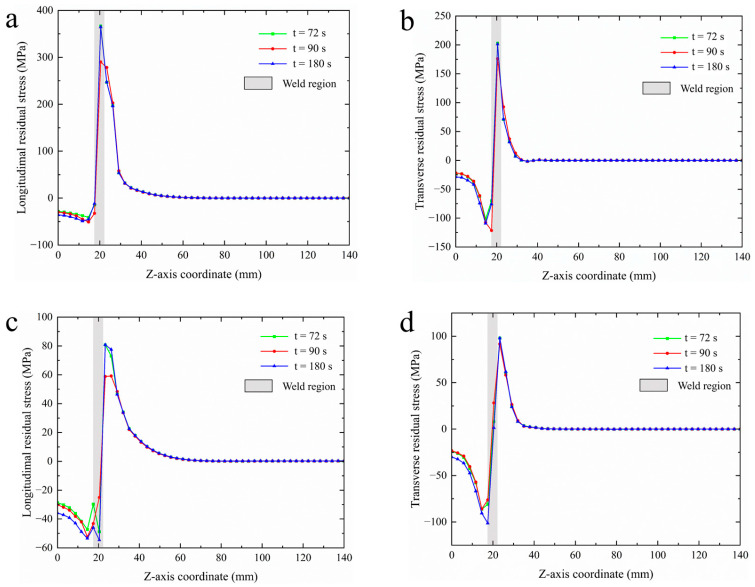
Residual stress distribution curves for sampling lines L5, L6 under varying welding durations: (**a**) longitudinal residual stress for L5; (**b**) transverse residual stress for L5; (**c**) longitudinal residual stress for L6; (**d**) transverse residual stress for L6.

In the thickness direction (*z*-axis) of the welded components, differential residual stress distributions were analyzed using sampling lines L5 and L6, as illustrated in Figure 18. Overall, it was observed that regardless of the type of residual stress (e.g., longitudinal residual stress, transverse residual stress), the stress distribution patterns remained consistent across different welding durations, with residual stress concentrated in the weld and heat-affected zones, notably between the 10–30 mm region of the *z*-axis. Figure 18a shows the distribution curve of longitudinal residual stress for sampling line L5 across different welding durations, in which residual compressive stress appears at 40 MPa under the flange base due to applied boundary conditions and temperature gradients generated by heat diffusion during welding. As the sampling point approaches the weld, compressive residual stress climbs, peaking at 50 MPa in the near-weld zone, with a 90 s welding duration exhibiting slightly higher residual compressive stress than the other two durations. In the weld region, residual compressive stress rapidly transitions to residual tensile stress, peaking in the weld area. For welding durations of 72 s and 180 s, the residual tensile stress peaks hover around 350 MPa, while during a 90 s welding duration, this peak dips to nearly 300 MPa—a 14% reduction. As measurements are taken further from the center of the weld, tensile stress gradually weakens until residual stress levels approach zero away from the heat-affected area. Figure 18b displays the transverse residual stress distribution curve for sampling line L5. In contrast to Figure 18a, notable transverse residual compressive stresses occur in the heat-affected zone, measured at approximately 125 MPa, while transverse residual tensile stress peaks decline to around 200 MPa, with minimal differences across the three welding durations. In Figure 18c, the distribution curve for longitudinal residual stress along sampling line L6 exhibits comparable patterns for all three conditions, mainly concentrated near the weld and center of the weld. Notably, there is a significant fluctuation in residual compressive stress in areas of the flange, with a marked reduction in the peak of longitudinal residual tensile stress, reported at 80 MPa; during a 90 s welding duration, longitudinal residual tensile stress falls further, reaching 60 MPa. Figure 18d illustrates the transverse residual stress distribution curve for sampling line L6. Upon evaluating the distribution of transverse residual stress at sampling line L6, compressive residual stress is present in the flange region while residual tensile stress occurs in the shaft, showing alignment in peak values, both at 100 MPa. All residual stresses are specifically presented in Table 8. Due to the pronounced temperature variations at the weld center, compressive stress transitions to tensile stress, with stress peak values notably concentrated at the edges of the weld. In conclusion, selecting a welding time of 90 s appears the most appropriate for arc welding 45Mn steel flange shafts of this size.

In summary, while maintaining a consistent welding power, variations in welding duration have a minor overall impact on the residual stress distribution in welded components but a significant influence on the magnitudes of those stresses. For this experiment, the optimal welding duration for the 45Mn steel flange shaft was determined to be 90 s. Longitudinal residual stress values were found to be greater than the transverse residual stress, generally attributed to the stronger constraints on longitudinal thermal contraction imposed by the welding direction, leading to a more pronounced accumulation of stress. Conversely, although subjected to some constraints, the transverse stresses in circular welds were partially neutralized by the symmetrical structure, leading to relatively lower peak residual stress values. In addition, in welding processes, the intrinsic interconnections between temperature, deformation, and residual stress fields are clearly discernible; temperature gradients induce deformation via non-uniform thermal expansion, while constrained deformation, in turn, dictates the distribution characteristics of residual stresses. This multi-field coupled dynamic mechanism not only reveals the core laws governing welding thermos-mechanical behavior, but also provides a critical basis for optimizing welding processes and enhancing structural integrity.

## 5. Conclusions

In this study, we explored the application of 45Mn steel in large structural welding, using a flange shaft as a typical component for experimental testing and simulation modeling. By integrating empirical measurements with a fitted heat source model, conducting thermodynamic coupling simulations, and utilizing a thermoplastic constitutive model, the temperature field, deformation field, and residual stress field under various welding process parameters were analyzed. The main conclusions drawn from this research are as follows:

(1) A combined heat source model was developed based on the geometric characteristics of the weld pool. By extracting the actual weld fusion line profile and fitting a rotational heat source distribution, the model achieved a highly accurate simulation of the complex “crescent-shaped” weld pool. Compared to the traditional double-ellipsoidal model (33.3% error) and the homogeneous volumetric model (11.1% error), the proposed model reduced the weld pool depth error to only 2.2% without requiring complex parameter calibration. This presents a more efficient and reliable approach to heat source modeling for arc welding simulations.
(2) The evolution of temperature and deformation fields during the welding process was systematically investigated. The simulation revealed pronounced temperature gradients along the component thickness, with higher peak temperatures and faster heating rates occurring at regions closer to the weld seam. Deformation analysis demonstrated that, while the welding duration significantly influences the magnitude of deformation, it does not alter the overall deformation pattern. Among the evaluated conditions, a welding time of 90 s appears to be more suitable for large-diameter flange shafts, effectively reducing residual deformation. Furthermore, increasing the shaft diameter was shown to enhance the structural stiffness, thereby mitigating welding-induced distortion. Those findings offer valuable references for evaluating the risk of welding deformation and cracking, and support the development of preventive strategies to ensure structural integrity during fabrication.
(3) The simulation results of residual stress revealed that longitudinal residual stresses are generally higher than transverse residual stresses. Along the welding direction, stresses were primarily concentrated within the weld zone and adjacent heat-affected regions. The distribution trend remained consistent across different welding durations, characterized by an initial rise following arc ignition, a stabilized peak during steady-state welding, and a decline after arc termination. Notably, when the welding time was set to 90 s, the residual stress gradient perpendicular to the welding direction was reduced by 19%, indicating that a properly extended welding duration can effectively alleviate stress concentration and help minimize the risk of structural warping and distortion.
(4) Based on simulation results, a welding time of 90 s may be a preferable choice for arc welding of 45Mn steel flange shafts of this dimension.

Despite the meaningful findings of this study, certain limitations remain, providing directions for future research. First, as the study primarily focused on the macroscopic thermal behavior and stress evolution during welding, it did not delve into the microstructural transformations occurring in the heat-affected zone (HAZ), nor did it explore the application of non-destructive testing (NDT) techniques. Second, with respect to the influence of welding process parameters on microstructural development and fatigue performance, the study only offers preliminary insights based on simulation data, which require further experimental validation. These issues will serve as key areas for future investigation. In addition, given the growing industrial emphasis on integrating process simulation with non-destructive evaluation, future work will explore coupling finite element model outputs—such as thermal histories in critical regions—with NDT methods like laser thermography, thereby enhancing the framework for intelligent welding quality assessment and structural reliability analysis.

## Figures and Tables

**Table 1:** Chemical composition of base material and filler material (wt.%).

Material	C	Si	Mn	P	S	Ni	Cr	Cu	Mo	V	Fe
45Mn steel	0.46	0.24	0.71	0.016	0.006	-	0.0131	0.0097	-	-	Bal.
E5015	0.15	0.90	1.60	0.035	0.035	0.30	0.20	-	0.30	0.08	Bal.

**Table 2:** Welding process parameters.

Process Parameters	Single-Pass Welding
Power (W)	4080
Current (A)	170
Voltage (V)	24
Shielding Gas	CO_2_
Shielding Gas Flow Rate (L/min)	15
Welding Thermal Efficiency	0.8

**Table 3:** Mesh sensitivity analysis results (welding time: 180 s).

Mesh Size (mm)		Measuring Point	Peak Temperature (°C)	Time of Peak Temperature (s)
Refined Area	Non-Refined Area			
0.5	1	G	212	192
		H	161	485
		I	145	618
2	4	G	195	215
		H	148	520
		I	132	640
1	2	G	206	200
		H	157	500
		I	142	625
1	1	G	207	198
		H	158	495
		I	143	623
1	3	G	203	204
		H	154	505
		I	139	630
0.8	2	G	204	197
		H	156	498
		I	142	625
1.2	2	G	201	203
		H	155	503
		I	141	627

**Table 4:** Material property parameters for 45Mn steel and E5015 filler material in the temperature range of 25 °C to 1600 °C.

Material	Temperature (°C)	Density (g/cm^3^)	Thermal Conductivity (W/(m∙°C))	Specific Heat (J/(g∙°C))	Young’s Modulus (GPa)	Poisson’s Radio	Thermal Expansion Coeff. (10^−6^/°C)	Yield Stress (MPa)
45Mn steel	25	8.04	17.06	0.452	199.47	0.293	11.70	355.18
	100	8.00	17.98	0.475	192.90	0.297	24.53	300.03
	200	7.94	19.20	0.498	184.03	0.303	24.53	261.78
	400	7.83	21.62	0.534	165.90	0.315	24.53	226.48
	700	7.66	25.25	0.582	137.72	0.333	24.55	204.72
	1000	7.50	28.86	0.629	108.36	0.350	24.61	23.34
	1300	7.35	32.47	0.679	77.82	0.368	24.72	3.11
	1600	6.89	35.63	0.829	0	0.500	35.54	0
E5015	25	7.84	63.63	0.447	210.13	0.290	12.50	391.43
	100	7.82	59.82	0.478	206.81	0.292	12.68	327.69
	200	7.79	54.64	0.517	200.99	0.296	13.05	281.71
	400	7.72	44.30	0.629	182.92	0.304	13.87	240.02
	700	7.61	33.69	0.945	142.93	0.316	15.09	105.80
	1000	7.55	28.90	0.625	105.71	0.352	13.12	22.69
	1300	7.39	32.47	0.674	74.762	0.370	15.99	5.73
	1600	6.93	34.96	0.825	0	0.500	27.79	0

**Table 5:** Peak temperature comparison between simulation models and experimental data at measuring points (welding time: 180 s).

Measuring Point	Type	Peak Temperature (°C)
G	Combined heat source	206
	Uniform volumetric heat source	221
	Double-ellipsoidal heat source	216
	Experimental	210
H	Combined heat source	157
	Uniform volumetric heat source	145
	Double-ellipsoidal heat source	163
	Experimental	160
I	Combined heat source	142
	Uniform volumetric heat source	148
	Double-ellipsoidal heat source	128
	Experimental	145

**Table 6:** Summary of maximum and minimum residual stresses for sampling lines L1 and L2 under different welding times.

Sampling Line	Type (Welding Time)	Max Residual Stress (MPa)	Min Residual Stress (MPa)
L1	72 s	293.57	240.52
	90 s	259.48	190.95
	180 s	293.92	230.33
L2	72 s	372.4	320.74
	90 s	325.34	279.85
	180 s	381.46	316.25

**Table 7:** Summary of maximum and minimum residual stresses for sampling lines L3 and L4 under different welding times.

Sampling Line	Type (Welding Time)	Max Longitudinal Residual Stress (MPa)	Min Longitudinal Residual Stress (MPa)	Max Transverse Residual Stress (MPa)	Min Transverse Residual Stress (MPa)
L3	72 s	880.2	−58.5	833.4	−98.3
	90 s	670.2	−60.6	581.7	−83.9
	180 s	860.6	−46.9	825.3	−109.5
L4	72 s	1.8	−49.2	−2.8	−40.4
	90 s	1.9	−51.4	−4.1	−41.2
	180 s	1.3	−57.8	−5.1	−45.8

**Table 8:** Summary of maximum and minimum stresses for sampling lines L5 and L6 under different welding times.

Sampling Line	Type (Welding Time)	Max Longitudinal Residual Stress (MPa)	Min Longitudinal Residual Stress (MPa)	Max Transverse Residual Stress (MPa)	Min Transverse Residual Stress (MPa)
L5	72 s	366.6	−41.0	202.9	−101.7
	90 s	290.2	−50.8	176.0	−121.5
	180 s	364.7	−48.8	201.2	−109.1
L6	72 s	80.8	−48.9	98.1	−86.3
	90 s	59.2	−52.4	91.7	−85.9
	180 s	80.9	−54.6	98.0	−101.3

## Data Availability

The original contributions presented in this study are included in the article. Further inquiries can be directed to the corresponding author.

## Nomenclature

q	Heat flux density
η	Welding thermal efficiency
U	Welding voltage
I	Welding current
V	Heat source effective volume
$Q_{m}$	Welding heat input
ρ	Filler wire density
$r_{w}$	Filler wire radius
ω	Wire feed speed
$H_{d}$	Filler wire fusion enthalpy
ρ(T)	Temperature-dependent density
$c_{p}(T)$	Temperature-dependent specific heat
k(T)	Temperature-dependent thermal conductivity
$f_{r}$	Energy distribution fraction in the front half of the ellipsoidal heat source
$a_{2}$	Length of the back half of the ellipsoidal heat source
c	Depth of the ellipsoidal heat source
$\sigma_{1,2,3}$	The normal stresses acting along three directions that are orthogonal to each other, respectively
${\left\{d\varepsilon \right\}}_{p}$	Plastic strain increment
$\frac{\partial \bar{\sigma }}{\partial \left\{\sigma \right\}}$	Partial derivative of the scalar function $\bar{\sigma }$ with respect to the vector function $\left\{\sigma \right\}$
t	Welding time
$Q_{v}$	Heat source energy density
$h_{c}$	Heat transfer coefficient
ε	Emissivity
$\sigma_{B}$	Stefan–Boltzmann constant
$T_{0}$	Initial environmental temperature
$\varepsilon_{\mathrm{total}}$	Total strain
$\varepsilon_{\mathrm{elastic}}$	Elastic strain
$\varepsilon_{\mathrm{plastic}}$	Plastic strain
$\varepsilon_{\mathrm{thermal}}$	Thermal strain
$\varepsilon_{\mathrm{creep}}$	Creep strain
$\varepsilon_{\mathrm{phase}}$	Phase transformation strain
$f_{f}$	Energy distribution fraction in the rear half of the ellipsoidal heat source
$a_{1}$	Length of the front half of the ellipsoidal heat source
b	Width of the ellipsoidal heat source
$\bar{\sigma }$	Equivalent stress
$\sigma_{s}$	Uniaxial tensile yield limit
dλ	Plastic multiplier
